# Systematic Review on Neurotoxic Implications of Lead-Induced Gene Expression Alterations in the Etiology of Alzheimer’s Disease

**DOI:** 10.1007/s10571-025-01613-6

**Published:** 2025-11-07

**Authors:** Aluru Parithathvi, P. Harshitha, Kamalesh Dattaram Mumbrekar, Herman Sunil Dsouza

**Affiliations:** Department of Radiation Biology and Toxicology, Manipal School of Life Sciences, Manipal Academy of Higher Education, Manipal, Karnataka 576104 India

**Keywords:** Lead neurotoxicity, Neurodegeneration, Alzheimer’s disease, Amyloid plaques, Neurofibrillary tangles

## Abstract

Lead (Pb) is a hazardous heavy metal frequently used because it is readily available and inexpensive. Due to contaminated soil, dust, and items like paints and batteries, lead exposure is still an issue of concern in many nations. There is no known safe threshold of exposure, and it can have serious adverse effects on human health. Exposure to lead has been linked to detrimental effects on the developing nervous system of both children and adults. Alzheimer's disease (AD) is the most prevalent type of dementia affecting adults over the age of 65, resulting in a decrease in memory and thinking skills. In this review, we describe the role of lead in exacerbating the build-up of hyperphosphorylated tau proteins and formation of amyloid-β (Aβ) plaques, major neurotoxicants which can impair neuronal function leading to AD. We highlight the effect of developmental and lifelong lead exposure on various gene expression changes resulting in the formation of the neurotoxicants responsible to AD. Understanding the mechanisms related to Aβ plaques and neurofibrillary tangles (NFTs) formation serves as a novel approach to identify biomarkers for lead-induced AD and developing therapeutic interventions. Lead exposure has been related to adverse effects on the developing neurological systems of both adults and children.

## Introduction

Lead is a naturally occurring element known for its toxic properties which frequently form compounds with sulfur. It has been widely used since prehistoric times due to its availability, ductility, high density, minimal chemical reactivity, simple extraction, and low cost (Garza et al. 2006). Given its wide range of applications and several beneficial qualities, lead's ubiquity in nature raises serious concerns for worldwide public health (Ramírez Ortega et al. 2021). Many young children in third-world nations have been exposed to lead-contaminated dust and soil from battery mining and recycling (Lead Poisoning, WHO 2023). India, Nigeria, China, Indonesia, Philippines, Pakistan, Peru, France, Brazil, Mexico, and the United States are among the nations with a high incidence of lead poisoning in humans (Ramírez Ortega et al. 2021). Lead can cause multiple irreversible harms and has no biological purpose; thus, there is no safe exposure. However, it has been further reduced to 5 µg/dL as various detrimental effects were observed even at low levels (Lead poisoning, WHO 2023). According to the World Health Organization (WHO), there are no safe levels of lead due to its adverse impact on children even at very low levels (Lead Poisoning, WHO 2023) . Abnormal levels of lead in the diet have been associated with myeloma, leukemia malignancies, all types of lymphomas such as ovarian, lung, stomach, renal small and large intestine (Reddy et al. 2004).

### Prenatal and Early-Life Exposure

Exposure during pregnancy and early infancy may be more damaging since these are key developmental periods, when neurological and physiological developmental processes are particularly sensitive to environmental exposures such as lead (Halabicky et al. 2024). Lead passively diffuses across placental cell membranes without restriction, and the content of lead in maternal and fetal blood is highly correlated (Ettinger et al. 2007). The fetus produces 1,25-Dihydroxycholecalciferol, which regulates calcium transport in the placenta and is most active in the final trimester of pregnancy. It is a key phase due to the development of neuronal structures, synaptogenesis, inflammation, and brain enlargement, as well as the blockage of neurotransmitter synapses by lead (Rísová 2019). Lead transport is thought to be closely related to calcium ions (Ca^2+^) mobility in syncytiotrophoblasts. Lead in maternal and umbilical blood alters calcium transport to syncytiotrophroblasts (Lafond et al. 2004). During pregnancy and lactation, the skeleton may serve as an internal storage since lead from the bones is mobilized more during the postnatal phase than during pregnancy. Inadequate Ca^2+^ consumption in the diet may be connected to increased release of skeletal lead into the blood during the postnatal period (Rísová 2019). Around 95% of lead is deposited in these mineralized tissues and bone lead has a half-life ranging from years to decades, women and newborns will still be at risk for exposure even after ambient lead sources have been minimized (Ettinger et al. 2007). Lead which is getting deposited in the bones and due to its prolonged half-life, poses a significant risk of a high maternal body load considerably in advanced stages of conception (Dórea 2019).

Early-life exposure to neurotoxic chemicals is linked to an increased risk of neurodevelopmental outcomes. The infant load of each given chemical is ultimately determined by its unique chemistry, exposure, and metabolism. Nevertheless, children are simultaneously exposed to multiple dangerous metals (Dórea 2019). In addition, endogenous lead exposure is caused by an increase in bone resorption (Muciño-Sandoval et al. 2021). The major source of lead in children is due to their age-appropriate hand-to-mouth activity, children ingest nonfood items (pica) that might contain lead deposited on them in the form of dust or lead-based paint (Lead Poisoning, WHO 2023). BLLs even as low as 3.5 µg/dL is detrimental in children and need to be monitored (Lead poisoning, WHO 2023). Children living in older homes with lead-based paint have long been related to higher BLL. Soil, water and air in and around the lead-based industries and high traffic roads/highways have higher amount of lead. Children who dwell in locations with significant lead-based industrial and traffic pollution have higher lead levels, which can be linked to behavioral and cognitive impairments (Hai et al. 2018).

### Adult Lead Exposure

The majority of adult cases of high BLL are associated with occupational or recreational exposure, which may result in harm to different systems in the body. Early signs of lead toxicity include fatigue, gastrointestinal issues, poor appetite, irritability, headache, sleeplessness, metallic taste in the mouth, reproductive health issues, hypertension, lack of focus, depression, and muscular and/or joint pain (Lead poisoning, WHO 2023). The lowest lead level of concern is 5 µg/dL since it is thought that harmful health consequences happen at very low levels (Lead poisoning, WHO 2023). The U.S. Department of Health and Human Services (HHS) and Occupational Safety and Health Administration (OSHA) have issued second and third threshold level indicators i.e., 25 µg/dL and 40 µg/dL (CDC 2024a). Disability-adjusted life years (DALYs) for a disease or health condition in each age group are computed by adding years lost to early mortality and years lived with disability. According to the World Health Organization (WHO), lead exposure is projected to cause 21.7 million years of disability and death (DALYs) worldwide. This is equivalent to 3% of the global burden of chronic kidney illness, 30% of the global burden of idiopathic intellectual disability, and 4.6% of the global burden of cardiovascular disease (Lead poisoning, WHO 2023). Despite India's 2001 phase out of leaded gasoline, Kumar et al. reported that 31% of floor dust samples and 14% of windowsill samples had lead levels over permissible limits (Kumar et al. 2009). Traditional Chinese and Indian treatments also contain trace amounts of lead because of the historical perception that the metal has therapeutic benefits (Obeng-Gyasi 2019). Lead-containing unbranded medicines are used to treat diabetes, spleen, liver, and skin disorders (CDC 2024b). Lead accumulation in municipal landfills is mostly caused by the disposal of lead-contaminated waste, the removal of lead-based paint from buildings and bridges, and the recycling of batteries in industry. Lead strongly combines with soil particles and is found in the top layer of soil (Gupta et al. 2008). Studies have reported high levels of lead in pesticides and herbicides which further deposit in the plants (Alengebawy et al. 2021; Defarge et al. 2018). Lead is also widely used in the production of paint, fuel, batteries, food cans, ceramics, jewelry, cosmetics (kohl, sindoor, lipsticks) (Al-Saleh et al. 2009; Ramírez Ortega et al. 2021; Shah et al. 2017). Long-term sources include gunshot wounds sustained as a result of a bullet injury, either accidentally or for criminal motives. Elevated BLLs associated with bullet injuries were observed among young adult males in non-occupational settings (Dsouza et al. 2011; Weiss et al. 2017). Inorganic lead exposure is a significant public health concern as it can have cognitive effects even at minimal concentrations. The aged population is more frequently exposed to the effects of lead exposure, which might cause aberrant cognitive aging performance (Ramírez Ortega et al. 2021).

## Methods

**Fig. 1:**
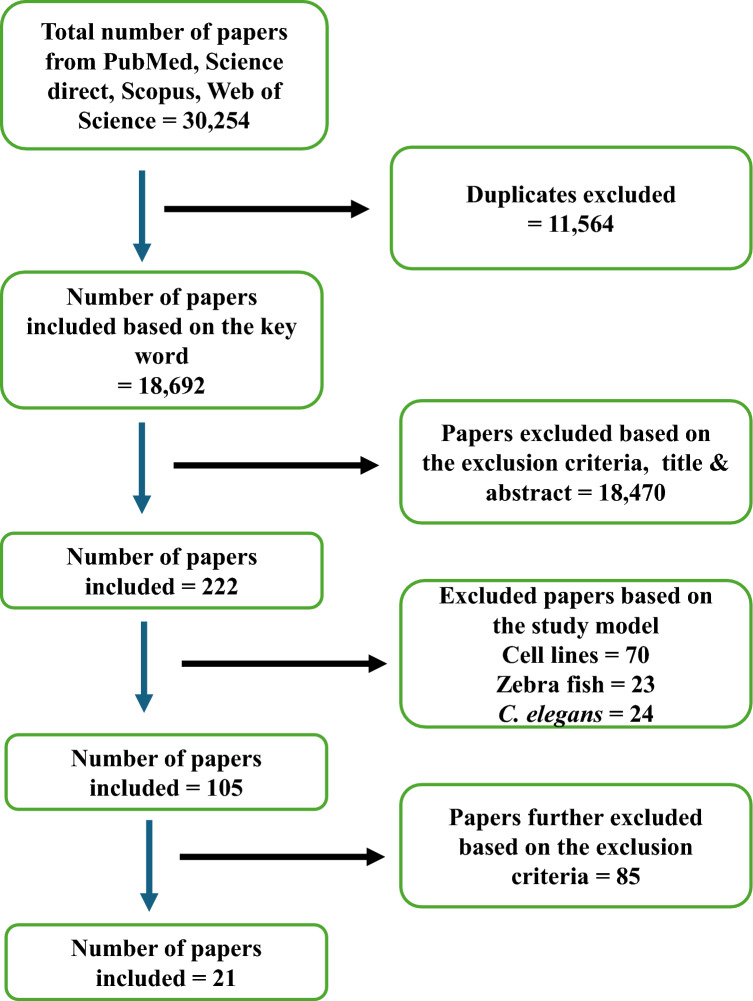
Fig. 1 describes the systematic selection of papers from different databases based on the keywords, inclusion and exclusion criteria

To investigate the relationship between lead exposure and neurotoxicity during the prenatal, perinatal, postnatal, and continuing adult stages, a literature search was conducted from 2000 to 2024 using the search engines "Science Direct," "Web of Science," "Scopus," and "PubMed." The search was conducted using the individual keywords “Alzheimer's disease,” “amyloid protein”, “tau protein” and “lead neurotoxicity”. A total of 30,254 papers were obtained based on the keywords, and 21 papers were shortlisted further as per the inclusion and exclusion criteria (Fig. 1). The PRISMA guidelines were followed to construct the review paper.

### Inclusion Criteria

All studies that evaluated the role of lead in causing AD were identified based on the title, abstract, and full text with keywords. This paper presents studies on prenatal, early and entire life lead exposure in human, animal models (rats, mice, primates)*, C. elegans* and zebrafish.

### Exclusion Criteria

Lead neurotoxicity causing neurodevelopmental and neurological disorders other than AD were excluded and in vitro studies were not considered*.* AD and neurological changes caused due to co-occurrence of lead and other metals/chemicals. Duplicate and review papers were excluded.

## Pathophysiology of AD

AD is a neurodegenerative disease, one of the most prevalent types of dementia and one of the primary reasons for cognitive impairment. This is a progressive brain disorder, triggering the death of brain cells leading to gradual loss of memory and thinking skills. This neurological disorder primarily affects adults over the age of 65, influencing cognitive functions such as comprehension, attention, judgment, and reasoning skills (Kumar et al. 2024). AD attributes to 5–10% of dementia cases, with frontotemporal lobar degeneration, Lewy body dementia, Parkinson's disease with dementia, normal pressure hydrocephalus, and vascular dementia, the other most prevalent causes of dementia. Vascular dementia and Lewy body dementia are predominantly associated with mixed pathology, including concurrent AD (Abubakar et al. 2022; Dementia, WHO 2023). Around 10 million cases of dementia are recorded each year, with over 60% of the people living in low- and middle-income countries (Dementia, WHO 2023). The clinical characteristics of AD comprise the accumulation of Aβ in the extracellular matrix of the brain and the development of intracellular neurofibrillary tangles, in addition to the usual neuronal loss and degeneration that results in memory loss and trouble learning (Chong et al. 2018). Individuals with AD experience cognitive impairment due to the progressive accumulation of tau and Aβ proteins. Aβ accumulates as β- and γ-secretases breaks amyloid precursor protein (APP), producing oligomers that induce neuronal death (Abubakar et al. 2022). Aβ triggers cell death by disrupting synaptic transmission between neurons, and tau NFTs prevent the flow of vital nutrients and other components within neurons. The tau hyperphosphorylation induces the production of aggregates of tau protein, which results in the formation of neurofibrillary tangles, twisted paired helical filaments in AD due to the accumulation of extracellular Aβ. AD develops in the hippocampal region and then migrates to the cortex, where it grows further, while tau aggregates are formed inside neurons (Monteiro et al. 2023). The risk factors for AD include environmental and genetic variables, and the environmental risks include exposure to pesticides, heavy metals, and industrial effluents (Monteiro et al. 2023). The major genes involved in AD and their associations with lead are explained further in this review.

### Role of Amyloid-beta (Aβ) Proteins in the AD Pathogenicity

**Fig. 2:**
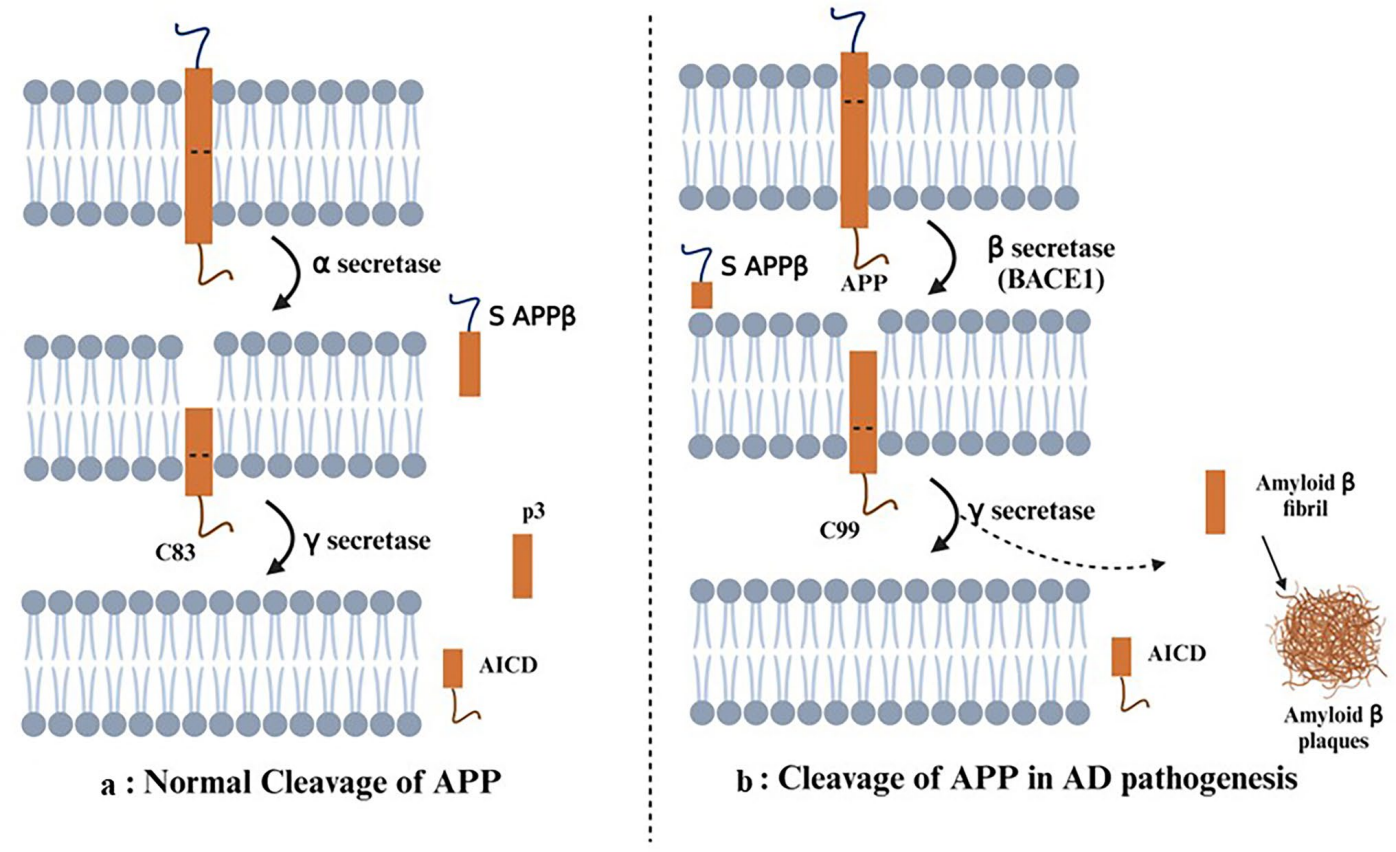
**a** describes the normal expression of APP protein in brain and its conversion into soluble Aβ by α and γ secretase. **b** describes the role of β secretase in incomplete cleavage of APP leading to formation of insoluble Aβ fibrils leading to the formation of amyloid plaques

To diagnose AD, neuritic plaques made of extremely insoluble Aβ must be present in the brain parenchyma. Aβ monomers can form various assemblages, such as protofibrils, oligomers, and amyloid fibrils. Soluble amyloid oligomers can spread throughout the brain, but amyloid fibrils are bigger, insoluble, forming amyloid plaques due to aggregation. In 1984, amyloid plaques and extracellular deposits yielded the initial amino acid sequence for Aβ (Chen et al. 2017). Brain neurons, blood cells, and astrocytes create Aβ, a 4 kDa fragment of APP (Hampel et al. 2021). Aβ peptides range in length from 39 to 43 residues, with the most common fragments being Aβ_1-40_ and Aβ_1-42_. The N-terminus of Aβ is hydrophilic, while amino acids 29-42 at the C-terminus are hydrophobic and comprise the transmembrane region of the amyloid precursor protein (Syme et al. 2004). The soluble extracellular domain of APP (sAPPα) is released during APP cleavage by α-secretase, although complete Aβ is not produced. A non-toxic p3 peptide is released when γ-secretase further cleaves the membrane-bound C-terminal region of APP (C83) (MacLeod et al. 2015). The APP cleavage through α-secretase has been described in Fig. 2a. In AD pathogenesis, the two membrane-bound endoproteases that cleave APP are β and γ-secretase. APP is cleaved twice by β-secretase at the ectodomain and γ-secretase at intramembranous locations, resulting in Aβ production (Hampel et al. 2021). Studies on the neuropathology and clinical phenotype of *Apolipoprotein E (APOE)* individuals indicate a strong correlation between Aβ metabolism and homeostasis. Studies have indicated that ApoE4 can help speed up the early nucleation or seeding of Aβ deposits, which in turn can help in the production of Aβ fibrils. Overexpressing ApoE4 in astrocytes, but ApoE3 was not observed to impair Aβ seeding and extend Aβ half-life in an aging animal model (Hampel et al. 2021; Murphy and LeVine 2010). In aqueous media, Aβ is a random coil peptide, but its amyloid fibrillar form has a high β-sheet structures. The three histidine imidazole rings and the N-terminus of Aβ are bound by copper ions (Cu^2+^), but not by tyrosine (Syme et al. 2004; Yugay et al. 2016). The apparent aggregation of Aβ at physiological Cu^2+^ and zinc ions (Zn^2+^) levels is a precursor to amyloid formation. As the pH of brain decreases in aged individuals, Cu^2+^-induced aggregation of Aβ occurs, and this slightly acidic environment replicates an aspect of inflammation observed in AD. Deposits of amyloid plaques have higher concentrations of certain metals like Cu^2+^_,_ Zn^2+^ and iron (Fe^3+^), yet their role in causing AD is unexplored (Syme et al. 2004). Extensive studies have enhanced our understanding of how the Aβ peptide is created and then broken down within the brain or carried out into the periphery, even if the role of APP itself remains unclear (Murphy and LeVine 2010). The detailed pathophysiology of Aβ in AD is described in Fig. 2b.

### Role of Tau Protein in the Pathogenesis of AD

Tau protein which is produced in neurons belongs to a group of proteins known as microtubule-associated proteins. Within the cytoskeletal network, microtubule assembly is strongly linked to tau protein (Garg et al. 2016). Tau proteins form intraneuronal filaments, which are essential for the development of various neurodegenerative diseases (Muralidar et al. 2020). Tau proteins have four functional domains i) N-terminal projection domain ii) proline-rich region (PRR) iii) microtubule-binding domain iv) C-terminal domain. The human tau gene, which is also known as *microtubule-associated tau protein (MAPT)*, is located on chromosome 17 at 17q21.31. It has 16 exons of which exon-1 is part of the promoter. In the human brain, the major tau protein is encoded by 11 exons. Alternating splicing of exons 2, 3, and 10 leads to six molecular isoforms of tau in the human brain (Duan et al. 2017; Muralidar et al. 2020). The 6 isoforms namely three 3R taus: 0N3R, 1N3R, 2N3R and three 4R taus: 0N4R, 1N4R and 2N4R. 0N indicates exclusion of E2 and E3, 1N and 2N indicate inclusion of only E2 and the E2 and E3, respectively. The biggest tau in the human brain is 2N4R tau which consists of 441 amino acids (tau-441). The specific tau isoform expressed in the fetal human brain is 0N3R; tau-352, which lacks both amino terminus inserts and an extra microtubule-binding repeat (Iqbal et al. 2010; Muralidar et al. 2020).

**Fig. 3:**
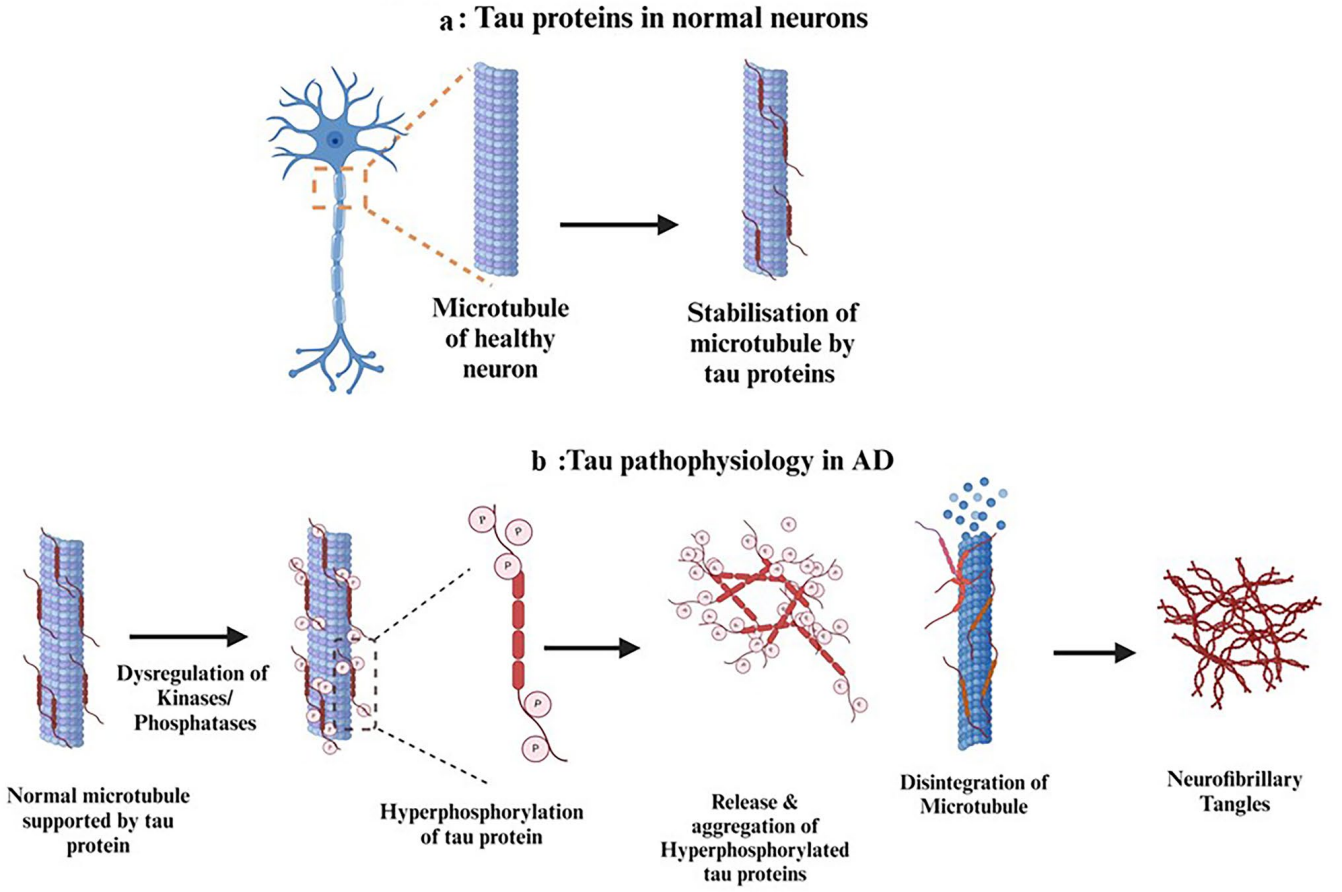
**a** describes the role of tau protein in stabilizing the microtubules in normal neurons. In **b**, kinases and proteases are disturbed, leading in hyperphosphorylation of tau protein, which dissociates from microtubules and disintegrates them. The hyperphosphorylated tau protein then aggregates to form NFTs

Post-translational alterations, particularly tau hyperphosphorylation, are thought to play a crucial role in AD by affecting microtubule assembly and generating tau aggregation (Chong et al. 2018; Iqbal et al. 2010). According to a 2017 study by Duan et al. tau mostly causes protofilaments to crosslink laterally to stabilize microtubules. The process of tau molecules binding across protofilaments, either in a lateral binding geometry or a combination of lateral and longitudinal binding, may result in this type of cross-linking. Tau-tau interactions between longitudinally bound tau molecules may serve as a substitute, but not mutually exclusive, means of reinforcing lateral connections between protofilaments. Distinct tau-microtubule-binding geometries could serve various purposes, such as controlling motor activity and microtubule spacing stabilizing the neurons (Duan et al. 2017). The binding of tau in normal neurons is described in Fig. 3a. The organization of microtubules is disrupted in AD patients due to the interference of tau misfolding with microtubule binding, preventing tau from being linked to tubulin or promoting microtubule assembly. Tau hyperphosphorylation causes conformational changes that contribute to the development of NFTs by aggregating tau monomers into oligomers (Chong et al. 2018; Iqbal et al. 2010). Phosphorylated tau protein is transported to distal locations in the neuropil because it has an affinity for kinesin. This could account for the observation that tangle illness in AD patients appears to begin distally and progress retrogradely to the perikaryon. Research indicates that the longest form of tau protein (441 amino acids) contains about 80 potential serine or threonine phosphorylation sites (Kolarova et al. 2012). All of these potential sites—aside from Ser-262, Ser-293, Ser-324, and Ser-356 in the R1, R2, R3, and R4 domains, respectively- are found in the proline-rich region around the microtubule-binding region (MTBR) and at the C-terminal extremity of the tau protein molecule. The abnormal phosphorylation of tau in this disease could be caused by either tau phosphatases downregulation or the overexpression of tau kinases; however, these two hypotheses are not exclusive. Glycogen synthase kinase 3β (GSK-3β), cyclin-dependent kinase-5 (cdk5), cAMP-dependent protein kinase (PKA), and calcium/calmodulin-dependent kinase II (CaMK-II) are among the enzymes that have been examined and are thought to be the most significant in the phosphorylation of tau in the brain (Dickey et al. 2007; Drewes et al. 1995). There is a discussion on the processes that cause tau protein to stop functioning. It is suggested that the primary reason for this failure is abnormal post-translational alterations. Numerous modifications, such as hyperphosphorylation, acetylation, nitration, ubiquitination, proteolytic truncation, conformational changes, glycation, and other processes, have been linked to the loss of normal function and the acquisition of pathological features in the tau protein (Martin et al. 2011; Min et al. 2010). The detailed pathophysiology of tau protein in AD is described in Fig. 3b.

## Neurotoxic Effect of Lead on the Genes Associated with AD

As discussed in the previous sections, Aβ and NFTs are key neurotoxicants in AD pathogenesis, altering pathways that cause cognitive decline, motor dysfunction, and neurodegeneration (Min et al. 2010; Syme et al. 2004). Numerous studies have reported the influence of lead on various genes involved in elevated *APP* expression and tau hyperphosphorylation leading to Aβ and NFTs formation (Lanphear et al. 2000; Leão et al. 2021; Zhou et al. 2018). This section discusses the effect of lead on various genes involved in Aβ and tau influencing the onset of AD are described in Table 1.

**Table 1:** List of genes associated with AD and impact of lead on specific genes

S. No	Gene Name	Location	Protein	Role in AD	Influence of lead	References
01	*App (Mice)*	Chromosome 21 (21q21.2–3)	Amyloid precursor protein	Overexpression shows a positive effect on cell health and growth leading to formation of plaques	Upregulation and early expression of *APP* gene	O’Brien and Wong 2011; Zhou et al. 2022
02	*PSEN1*	Chromosome 14 (14q24.2)	Presenilin-1	*PSEN1* is involved in the cleavage of C-terminal transmembrane region of APP and the production of Aβ peptide	No reported study	Bagaria et al. 2022
03	*PSEN2*	Chromosome 1 (1q42.13)	Presenilin-2	Subunit of γ-secretase, involved in cleavage of APP protein forming Aβ peptide	No reported study	Cai et al. 2015
04	*APOE*	Chromosome 19 (19q13. 32)	Apolipoprotein E	Affects the deposition of Aβ forming senile plaques causing cerebral amyloid angiopathy and plays a major role in amyloid-β metabolism	No reported study	Liu et al. 2013
05	*Bace-1 (Mice)*	Chromosome 11 (11q23.3)	Beta-site APP cleaving enzyme 1	APP cleaving enzyme, responsible for initiation of Aβ formation	Upregulation of *Bace1* gene mRNA levels	Vassar et al. 2009
06	*TREM2*	Chromosome 6 (6p21. 1)	Triggering Receptor Expressed in Myeloid Cells 2	Involved in uptake of Aβ (Aβ_40_, Aβ_42_) by microglia	No reported study	Gratuze et al. 2018
07	*PLD3*	chromosome 19 (19q13. 2)	Phospholipase D3	PLD3 contributes to Aβ accumulation in AD by disrupting Aβ metabolism	No reported study	Rosene et al. 2021
08	*CLU*	Chromosome 8 (8p21. 1)	Clusterin	Involved in prevention of accumulation of Aβ by interacting with Aβ_1-40_, Aβ_1-42_	No reported study	Fareed et al. 2022
09	*ABCA7*	Chromosome 19 (19p13. 3)	Adenosine triphosphate (ATP)-Binding Cassette, Subfamily A (ABC1), Member 7	Uptake of Aβ_1-40_, Aβ_1-42_, regulation of cholesterol homeostasis and Aβ efflux at BBB	No reported study	De Roeck et al. 2019
10	*ADAM10*	Chromosome 15 (15q22)	ADAM metallopeptidase domain 10 (cell surface proteins)	Capable of proteolytic cleavage of APP that prevents Aβ production	No reported study	Elsworthy et al. 2022
11	*SORL1*	Chromosome 11 (11q23)	Sortilin related receptor 1	Regulator of the trafficking and processing of APP, destructing Aβ and interacting with tau and ApoE protein	No reported study	Yin et al. 2015
12	*PICALM*	Chromosome 11 (11q14.2)	Phosphatidylinositol binding clathrin assembly protein	Involved in pathways associated with Aβ clearance, APP trafficking, synaptic function, and endocytosis	No reported study	Khan 2016
13	*BIN1*	Chromosome 2 (2q14.3)	Bridging Integrator 1	Increases intracellular Aβ levels by interfering with BACE1 recycling, involved in tau hyperphosphorylation	No reported study	Saha et al. 2024
14	*SNCA*	Chromosome 4 (4q22.1)	Alpha-synuclein	Lewy body AD	No reported study	Linnertz et al. 2014
15	*C9ORF72*	Short (p) arm of chromosome 9, open reading frame 72	Chromosome 9 open reading frame 72	Role in AD is not clearly elucidatedInvolved in causing frontotemporal dementia	No reported study	Xu et al. 2021
16	*CD33*	Chromosome 19 19q13. 3	CD33 or Siglec-3 (sialic acid binding Ig-like lectin 3	Modulating the microglial activation and inhibit Aβ clearance	No reported study	Zhao 2018
17	*CR1*	Chromosome 1 (1q32)	Complement receptor 1	Mutations induce elevation of Aβ_42_ and accumulation of neurofibrillary tangles and hyperphosphorylation of tau	No reported study	Zhu et al. 2015
18	*FUS*	Chromosome 16 (16p11.2)	Fused in Sarcoma/Translocated in Lipo-Sarcoma	Associated with frontotemporal dementia	No reported study	
19	*Mapt(Mice)*	Chromosome 17 (17q21)	Microtubule Associated Protein Tau	Changes in MAPT gene induces β sheet aggregation in tau proteins inducing disintegration of microtubules of neurons	Prenatal lead exposure induces hyperphosphorylation in tau protein	Dash et al. 2016; Strang et al. 2019
20	*TARDBP*	Chromosome 1 (1p36)	TAR DNA-binding protein	Hyperphosphorylation of TDP-43 leads to formation of senile plaques in neurons. These plaques aggregate with amyloid plaques and NFT’s increasing the severity of the disease	No reported study	Meneses et al. 2021; Jo et al. 2020
21	*GRN*	Chromosome 17 (17q21-22)	Progranulin	Mutations in GRN gene were observed to cause AD – like pathophysiology but mostly associated with FTD	No reported study	Nalls et al. 2021; Perry et al. 2013
22	*LRRK2*	Chromosome 12 (12p11.2-q13.1)	Proteins with armadillo repeat region, an ankyrin repeat region, a leucine-rich repeat domain, a kinase domain, a RAS domain, a GTPase domain, and a WD40 domain	Lewy body AD	No reported study	Linnertz et al. 2014
23	*EPHA1*	Chromosome 7 (7q34-q35)	Ephrin Type-A Receptor 1	Disruption in ephrin signaling leads to neuroinflammation and immune dysregulation. The association between *EPHA1* gene and AD is poorly understood	No reported study	Barber 2012; Buhl et al. 2022
24	*MS4A*	Chromosome 11 (11q12)	class of tetraspanin proteins	Regulates solubility of TREM2	No reported study	Deming et al. 2019
25	*CASS4*	Chromosome 20 (20q13. 31)	Crk associated substrate 4	Crk associated substrate 4 plays a key role in focal adhesion, kinase regulation, cell adhesion, migration and motility. Despite the exact function is unknown, it has been associated with plaques and NFT’s formation in AD	No reported study	Beck et al. 2014; Giri et al. 2016
26	*VSNL1*	Chromosome 2 (2p24.2)	Visinin Like 1 protein	Visinin Like 1 protein, which is a neuronal Ca^2+^-sensor protein. It is associated with cognitive impairment, NFT’s and plaque formation due to its role in Ca^2+^ signaling and homeostasis	No reported study	Braunewell 2012; Dwary et al. 2012
27	*RTN*	Chromosome 14 (14q23.1)	Reticulon/ neuroendocrine-specific protein (NSP)	Reticulon- 3 was observed to inhibit BACE1 enzyme activity and is also involved in oligomerization of dystrophic neurites in AD	No reported study	Chiurchiù et al. 2014; Shi et al. 2017
28	*FGF*	Chromosome 3 (3q28-q29)	fibroblast growth factor homologous factor family proteins	FGF2 protein is involved in mitigation of tau pathology, and it also downregulates the BACE1 enzyme expression which is further involved in decreased formation of plaques	No reported study	Alam et al. 2022; Klimaschewski and Claus 2021
29	*PLXNA4*	Chromosome 7 (7q32.3)	Plexin-A4	Plexin-A4, in complex with neuropilin-2, acts as a receptor for Aβ, leading to tau phosphorylation and aggregation	No reported study	Chung et al. 2021; Wang et al. 2016
30	*Gsk3β(Mice)*	Chromosome 3 (3q13.33)	Glycogen synthase kinase-3 beta	Promotes tau hyperphosphorylation, and amyloid plaque aggregation leading to neurodegeneration	Lead was involved in phosphorylation of *Gsk3β*	Gąssowska et al. 2016; Lauretti et al. 2020

Developmental exposure in primates (*Macaca fascicularis) by* Bihaqi and Zawia (2013) reported a significant increase in tau protein levels, which included Thr (threonine)-181, Thr-212, Ser (serine)-235, and Ser-396, in the later ages of the progeny. The mRNA levels of the *Tau, Sp3, Sp1* (Specific protein 1/3), and *Cdk5* genes were higher in aged primates than in control monkeys. Furthermore, as compared to the aged controls, the lead-exposed group had distinguished levels of Ser/Thr protein phosphatase activity (Bihaqi and Zawia 2013). A different study on chronic lead exposure found that, in comparison to controls, rodent models with higher levels of *App* mRNA expression at later stages. A considerable increase in the Sp1 transcription factor's levels and an early increase in *Sp1* DNA binding in later stages was observed in microarray analysis of transcription factors. It was shown that exposure to postnatal lead increased the expression of *App* mRNA for a brief period in the first month following birth, then decreased to low levels after a year, and then increased once again, peaking at 20 months (Basha et al. 2005). Prenatal exposure to lead acetate significantly elevated the BLL in the offspring compared to the controls. Although there was a discernible shift in the mRNA levels in the cerebellum or forebrain cortex was not observed, the western blot analysis revealed increased levels of tau protein. The study reported elevated tau phosphorylation at Ser-396, Ser-199, and Ser-202 in the forebrain cortex and cerebellum, whereas it showed no effect in the hippocampus. It was also observed that lead is involved in GSK-3β phosphorylation at Tyr-216 by 28% in the cerebellum and 25% in the forebrain cortex, whereas Ser-9 was unchanged in both brain regions (Gąssowska et al. 2016). Early AD-like disease symptoms have been observed to be induced by developmental lead exposure in young rats since it increases amyloid plaque deposition and Aβ accumulation. The Aβ_42_ expression was significantly higher in the hippocampus and the cerebral cortex of the first and second exposed groups compared to the control groups. The cerebral cortex and hippocampus showed significantly elevated *App* and *Bace1* (Beta-site APP cleaving enzyme 1) mRNA and protein expression following lead exposure (Zhou et al. 2018). Another study published in 2022 by Zhou et al. found that the high and low-level lead-treated groups produced more RAGE (Receptor for Advanced Glycation End Products) in the choroid plexus and micro vessels than the control group. Lead exposure significantly reduced the lead-treated groups' synthesis of LRP-1 (Low-Density lipoprotein receptor-related protein 1) protein, which is essential for Aβ endocytosis in the liver. Based on the elevated inflammatory responses and cell death in the choroid plexus and brain micro vessels, this study demonstrated that lead exposure at embryonic ages damaged two highly vascularized brain areas (Zhou et al. 2022).

**Fig. 4:**
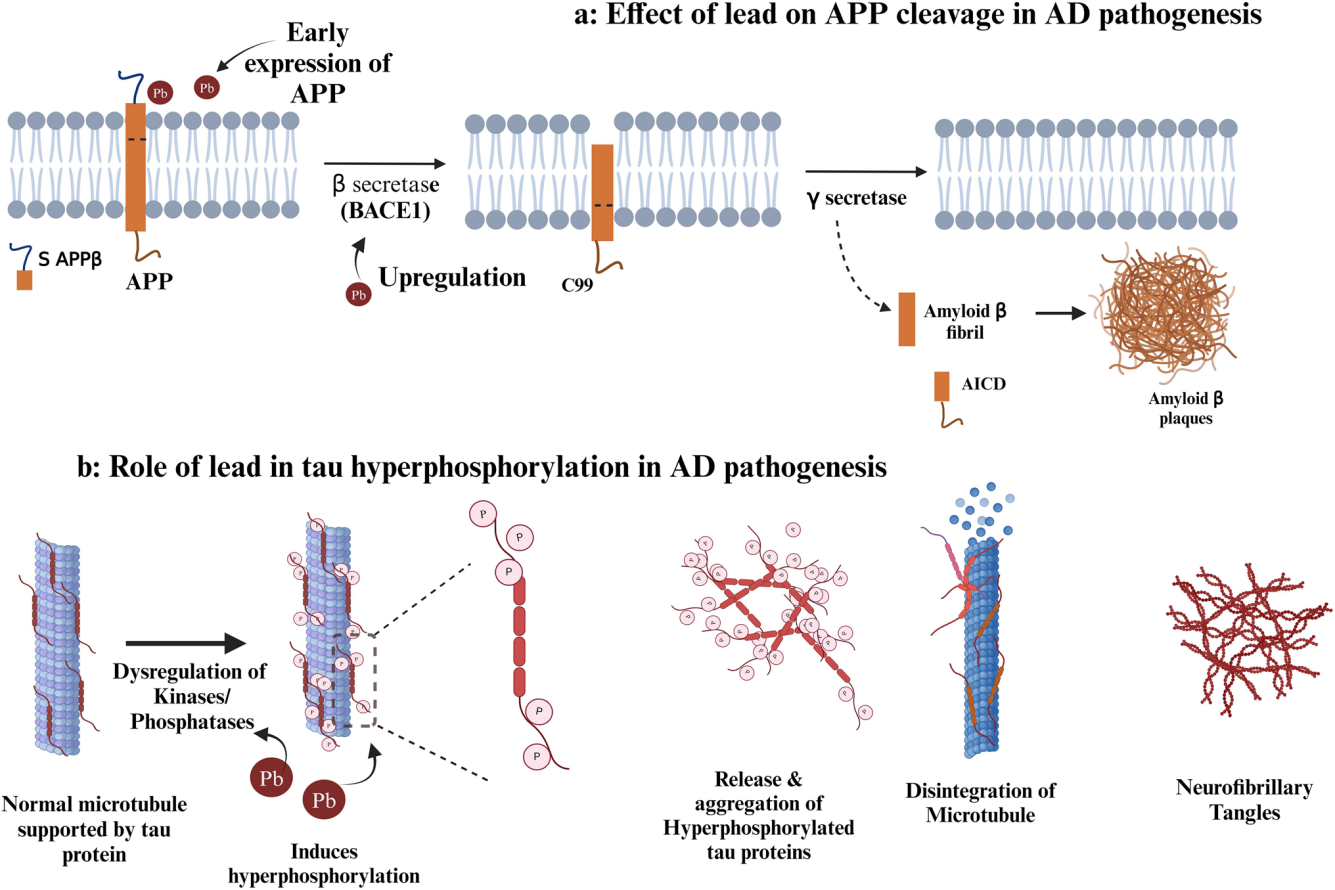
**a** depicts the role of lead in early expression of APP and upregulation of β secretase (BACE1) leading to incomplete cleavage of APP and formation of insoluble Aβ fibrils. **b** demonstrates the effect of lead in disrupting kinases and proteases involved in phosphorylation, which leads to hyperphosphorylation and the production of NFTs

A cohort study on prenatal lead exposure in a young adult population with a follow-up after 10 years revealed an elevation in Aβ_42_ levels in the adults having cord BLLs greater than 10 μg/dL compared to low and moderate cord BLLs. This study also showed an inverse relationship between cord BLL and the expression of genes associated with AD, such as *ADAM9, RTN4* and *LRPAP1* (Mazumdar et al. 2012). Liu et al. (2014) reported the lead exposure effect on Aβ at different life stages by exposing individuals to lead During Pregnancy (DP), During and After Pregnancy (DAP), and After Pregnancy (AP). Early-life lead exposure, even at low levels, is associated with significant changes in gene expression in later stages of life. While tau protein expression was relatively elevated in the lead-exposed group than in the control group, the levels of Aβ in the DP, DAP, and AP groups in this study were considerably higher in comparison to the control group (Liu et al. 2014). A study conducted by Zhang et al. (2012) observed an increase in hippocampal lead levels in rodents exposed to different doses of lead. This study also reported the hyperphosphorylation of tau protein at Ser-396 and Ser-404 in the hippocampus and the phosphorylation increased with increasing lead concentration (Zhang et al. 2012). Maternal lead exposure at different concentrations, such as 0.03, 0.1, and 0.3%, increased Aβ_40_ levels in the hippocampus on the 7th, 14th, and 21st days in progeny (Li et al. 2011). A similar study by Li et al. (2012), revealed an increase in hyperphosphorylated tau protein levels in the mouse hippocampus on the 21st day of life after maternal lead exposure, in addition to elevated tau protein expression levels, which impact learning, memory, and neurotransmission (Li et al. 2012) (Fig. 4).

Exposure to lead in early life contributed to elevated tau phosphorylation and Aβ levels by postnatal day (PND) 21 compared to those in the control groups. Administration of 0.1%, 0.5%, or 1% lead acetate through drinking water significantly increased Aβ and phosphorylated tau protein levels, whereas the 0.1% lead acetate group did not significantly change these levels (Li et al. 2010). Gu et al. (2011) exposed transgenic *App* mice to lead and subsequently generated hippocampal neurons from 1-day old progeny. Hippocampal neurons were cultured and exposed to different concentrations of lead for 24 h. The study found that the treated group had higher levels of Aβ_40_ in hippocampal neurons and cerebrospinal fluid compared to the control group. Lead exposure in transgenic mice significantly decreased the level of LRP-1 in the hippocampus and cerebellum, which was not observed in other regions of the brain (Gu et al. 2011). A study conducted in lead-exposed animal model to show the effect of early-life lead exposure through breast feeding reported significant decrease in the expression of miR-106b, which is involved in binding to *App* mRNA, on PND 700 (Masoud et al. 2016). Dash et al. (2016) observed that developmental lead exposure in transgenic *Mapt* mice significantly increased the hyperphosphorylation of tau protein at Thr-181 and Ser-396 in various cerebral cortical areas with age. *Tau* mRNA levels were also upregulated on PND 20. Cdk5 levels were elevated in lead-exposed mice at PND 20, 40, and 60, regardless of exposure duration. Prenatal lead exposure increased the BLL, accelerated Aβ_1-42_ deposition in mouse cortexes, and altered the levels of the Zonula Occludin-1 (ZO-1) and claudin 5 (CLDN5) proteins. Reduced protein and mRNA expression of LRP-1, increased protein and mRNA expression of App and Bace1, and enhanced tau phosphorylation were seen in both transgenic and lead-exposed animal models. The study revealed that following lead exposure, the count of activated astrocytes elevated in the transgenic mice brain, and these astrocytes clustered around the area of Aβ_1-42_ deposition. This sheds light on the process of preventing lead-induced AD through cerebrovascular pathways (Wu et al. 2020).

## Epigenetic Alterations Due to Lead-Induced Neurotoxicity

Dash et al. 2016 observed that developmental lead exposure in transgenic MAPT mice increased hyperphosphorylation of tau protein at Thr 181, and Ser 396 in various cerebral cortical areas with age. Tau mRNA levels were also shown to be upregulated on PND 20. The authors of this study discovered that lead exposure resulted in an equivalent and significant increase in miR-34c, which is associated with Mapt expression, between PND 20 and PND 50 (Dash et al. 2016). Early life lead exposure through breast feeding was observed to decrease the expression of miR-106b, which is involved in binding to AβPP mRNA on postnatal day 700. MiR-124 that binds to SP1 mRNA, has also been lowered in the lead-exposed animals compared to the control group on PND 700 (Masoud et al. 2016). Wen et al. 2022 investigated particular miRNAs related with lead exposure in a human population to investigate the link between lead and AD. This study discovered four miRNAs that were substantially linked with lead exposure: hsa-miR-3651, hsa-miR-150-5p, hsa-miR-664b-3p, and hsa-miR-627. These miRNAs were tested for expression in human brain tissue, and two of them were shown to be differentially expressed in AD patients' brains compared to controls. The analysis also identified gene targets for these miRNAs that were overrepresented in suspected AD-related pathways. In addition, the analysis looked at transcription factors that regulate miRNAs and gene targets. Wen et al. 2022 findings shed light on the possible utility of miRNAs as biomarkers for the risk of acquiring AD as a result of lead exposure (Wen et al. 2022).

Knowledge on the mechanism of various genes involved in formation of Aβ and hyperphosphorylated tau protein allows us to understand the AD pathogenesis. In this review we highlight the elevated levels of mRNA expression of LRP-1, App, Bace1, Mapt and Cdk5 genes, Sp1, Sp3, GSK3β, protein phosphatases and CLDN5 proteins that can be used as novel markers in diagnosis of AD.

## Preventive Measures of Lead Toxicity

Preventive approaches for lead toxicity focus on reducing environmental, nutritional, and occupational exposures through a combination of public health interventions and individual-level practices. To restrict lead absorption, strategies include the elimination of lead-based paints, the use of certified water filtration systems, the implementation of strong industrial emission regulations, and the promotion of calcium, iron, and vitamin C-rich diets (WHO, Lead Poisoning 2024). Prenatal exposure guidelines advocate routine BLL screening among at-risk pregnant women, avoidance of known sources of lead contamination, occupational exposure minimization, and focused dietary treatments to protect fetal development (CDC 2024a). Regular BLL monitoring in early childhood, particularly at 12 and 24 months, is crucial, as is strong lead-free regulation of consumer products (CDC 2024a).

WHO emphasizes that no level of lead exposure is considered acceptable and advocates for aggressive exposure reduction efforts (Lead poisoning, WHO 2023). The OSHA guidelines recommend an 8-h time-weighted average of 50 µg/m^3^ for air lead concentrations in workplaces. Medical monitoring is necessary for workers exposed to levels over 30 µg/m^3^ (CDC 2024a, 2024b). Preventive measures in the workplace include engineering controls, the use of personal protective equipment (PPE), strict cleanliness requirements, regular health monitoring, and ongoing worker education. Effective prevention of lead toxicity in vulnerable groups still requires a multidisciplinary, all-encompassing strategy that includes early detection, occupational safety, nutritional support, and environmental regulation (CDC 2024a, 2024b).

## Therapeutic Interventions

Several therapeutic strategies have been explored to counteract lead-induced neurotoxicity, focusing on antioxidant, anti-inflammatory, and chelating properties. Antioxidants like curcumin, Coenzyme Q10, and melatonin help reduce oxidative stress and improve neuronal health (Lu et al., 2022; Neha et al. 2025). Anti-inflammatory agents such as sodium para-aminosalicylic acid (PAS-Na) and 3-hydroxy-3′,4′,5′-trimethoxyflavone (HTMF) alleviate neuroinflammation and cognitive deficits. Chelating agents like succimer (DMSA), penicillamine effectively lower lead levels and mitigate oxidative damage, with betavulgaris juice (BVJ) enhancing these effects when combined with DMSA (Stangle et al. 2004). Additionally, neuroprotective compounds such as 5,7-dihydroxy-3′,4′,5′-trimethoxyflavone (TMF) and tanshinone IIA (TSA) have shown promise in reversing cognitive and motor impairments caused by lead exposure (Singh et al. 2024). However, the use of natural chelators during the exposure can reduce the risk of lead neurotoxicity.

## Conclusion

Lead damages the neurological system in humans through several different processes. Numerous studies have examined the role of lead in affecting behavior, IQ, and other aspects of neurodevelopment. Due to the late expression of the markers and symptoms of neurodegeneration, lead intoxication may pose a serious risk. However, relatively few research has investigated the influence of lead on neurodegeneration and related diseases. Lack of studies on how lead affects every gene connected to AD makes it difficult to diagnose the condition and identify biomarkers that contribute to lead-induced AD. Lead-induced neurotoxicity accelerates AD by promoting Aβ deposition and tau hyperphosphorylation, leading to cognitive decline, memory loss, and behavioral impairments. Clinical strategies can include routine blood-lead screening, early biomarker monitoring, and targeted interventions to mitigate neurodegeneration and neuroinflammation. Identifying the prognostic markers in blood correlating the brain parameters can help in early detection of lead-induced AD. Besides, the high clearance rate of Aβ plaques and tau protein makes the diagnosis more difficult in the early stages. Further investigation into the mechanism underlying lead-induced AD is crucial since lead has the potential to cause harm that persists across generations. Thus, understanding the mechanism by which lead affects different genes associated with AD can help in identifying new biomarkers that can be valuable in the early detection of the disease and in developing a putative therapeutic strategy for treating AD. Stringent public health policies and monitoring will help in reducing lead-induced neurotoxicity.

## Data Availability

No datasets were generated or analysed during the current study.
